# Sugary drinks taxation: industry’s lobbying strategies, practices and arguments in the Brazilian Legislature

**DOI:** 10.1017/S136898002100149X

**Published:** 2022-01

**Authors:** Aline Brandão Mariath, Ana Paula Bortoletto Martins

**Affiliations:** 1 Chamber of Deputies, Brasília, Brazil; 2 Post-graduate Program in Public Health Nutrition, School of Public Health, University of São Paulo, São Paulo, Brazil; 3 Center for Epidemiological Studies in Health and Nutrition, University of São Paulo, São Paulo, Brazil

**Keywords:** Corporate political activity, Lobbying, Public policy, Sugar-sweetened beverages, Taxation

## Abstract

**Objective:**

To assess the strategies, practices and arguments used by the industry to lobby legislators against sugary drinks taxation in Brazil.

**Design:**

We performed a content analysis of arguments put forward by sugary drink and sugar industries against sugary drinks taxation, using the framework developed by the International Network for Food and Obesity/Non-Communicable Diseases Research, Monitoring and Action Support to assess corporate political activity of the food industry.

**Setting:**

Two public hearings held in 2017 and 2018 in the Brazilian Legislature.

**Participants:**

Representatives from two prominent industry associations – one representing Big Soda and the other representing the main sugar, ethanol and bioelectricity producers.

**Results:**

The ‘Information and messaging’ and ‘Policy substitution’ strategies were identified. Five practices were identified in the ‘Information and messaging’ strategy (four described in the original framework and an additional practice, ‘Stress the environmental importance of the industry’). Mechanisms not included in the original framework identified were ‘Stress the reduction of CO_2_ emissions promoted by the industry’; ‘Question the effectiveness of regulation’; ‘Suggest public-private partnerships’; ‘Shift the blame away from the product’ and ‘Question sugary drinks taxation as a public health recommendation’. No new practices or mechanisms to the original framework emerged in the ‘Policy substitution’ strategy.

**Conclusions:**

The strategies and practices are used collectively and complement each other. Arguments herein identified are in line with those reported in other countries under different contexts and using different methodologies. Future research should address whether and under what conditions lobbying from this industry sector is effective in the Brazilian Legislature.

In the fight against the obesity and diet-related non-communicable diseases (NCDs) epidemic, regulation of ultra-processed food industry activities and practices has been claimed necessary as self-regulation and voluntary codes have been proven insufficient^(1-4)^. Among the recommended food industry regulations lies sugary drinks taxation^(5,6)^. Such fiscal policy is expected to influence consumers towards healthier beverage choices and to reduce added sugars intake, leading to better population health outcomes and a reduction in healthcare costs. In addition, it could increase government revenues to then be applied to public health systems and programmes^(1,7)^.

Increasing prices has been shown effective in reducing purchases and intake of sugary drinks both in experimental studies^(8,9)^ and impact analysis carried out in countries which have implemented this recommendation^(10-12)^. However, because it is a recent intervention, no population-wide studies of its effects on body weight and health are available yet. This public health measure has been adopted in countries such as Barbados, Belgium, Chile, Finland, France, Mexico, Norway, Saudi Arabia and Spain but also locally in the USA (Albany, Berkeley, Oakland, Philadelphia, *Navajo Nation* and, more recently, Seattle, San Francisco and Washington DC).

Not surprisingly, this type of regulation represents a threat to the ultra-processed beverage industry and therefore faces strong opposition. As a means to prevent being regulated, to postpone or to shape regulation in its favour, the industry uses a variety of corporate political activity (CPA) strategies and practices related to public health^(3,13-15)^. There is strong evidence that the sugary drink industry is highly engaged in CPA. Reported practices include, but are not limited to: sponsorship of public health organisations, research funding, lobbying against regulation, dissemination of messages to shift the focus away from sugary drinks as a cause of obesity, the use of corporate social responsibility actions and public relations campaigns, and the threat of using legal measures against government regulations^(16-24)^.

Although overall world sales of sodas have been decreasing since 2000, especially in North America, sales in the Middle East and Latin America are still on the rise^(25,26)^. The Pan American Health Organization estimates that the annual *per capita* sales of ultra-processed sugary drinks in Brazil have increased from 69·5 to 90·9 litres between 2000 and 2013^(26)^. With regard to sodas, the market is highly concentrated in the country, reflecting the global market. Estimates from 2015 account that Big Soda dominates 80% of the market share (Coca-Cola holds 61% and Ambev, which is responsible for producing and distributing PespiCo products in the country, holds 19%)^(27,28)^.

The consumption of sugary drinks, especially sodas, had been increasing over the years in Brazil, as reported in the main population-based surveys which assessed dietary intake. Surprisingly, for the first time, the most recent survey has shown a reduction in soda consumption. Results from the Household Budget Survey 2017–2018 have shown that the frequency of consumption has decreased from 23% to 15·4% in the last decade. The reasons for such seemingly positive finding are still unknown, but one possible explanation is the growing health concerns among more educated individuals, as the reduction was more pronounced in higher income households. Still, the mean *per capita* intake of soda was 67·1 ml/d (four-fold the intake of powdered drink mixes and industrialised juices and 4·5-fold that of dairy drinks). The intake of powdered drink mixes and industrialised juices has also reduced. The overall intake of dairy drinks has remained stable. Although it has increased in the highest income quartile, it has reduced in the others^(29)^. Despite this scenario, Brazil can still be considered a golden goose for the industry because of its tropical climate and its population (over 200 million inhabitants, the highest population in Latin America and the fifth worldwide).

Brazil was the first country to formally commit to the United Nations’ Decade of Action on Nutrition framework in 2017. At the time, one of the SMART commitments taken on by the Ministry of Health was to reduce the intake of sugar-sweetened beverages among adults by at least 30% by 2019^(30)^. Increasing taxes on sugary drinks would likely be the most appropriate policy to help achieve such a goal.

Under the Federal Constitution, legislation on the taxation system is an attribution of the Brazilian Legislature. Therefore, in order to increase taxes on sugary drinks, new law must be passed by the National Congress (which in Brazil is bicameral and comprises the Federal Senate and the Chamber of Deputies) and then sanctioned by the President^(31)^. Legislative proposals to increase taxes on sugary drinks have only recently been introduced (the first one was introduced in the Federal Senate in 2016 and has already been archived), and the discussion of the subject in the legislature is still incipient^(30)^.

We aimed to assess the strategies, practices and arguments used by the industry to directly lobby legislators against sugary drinks taxation in the country. This is the first study to address the CPA of the sugary drink industry in the Brazilian Legislature. To our knowledge, the only previous study which used the CPA framework developed by Mialon *et al*.^(14)^ to assess lobbying arguments against sugary drinks taxation was that carried out by Tselengidis and Östergren^(32)^, who analysed arguments publicised on the websites of stakeholders in the European Union.

## Methods

There have been two recent public hearings to discuss sugary drinks taxation as a public health policy in the Chamber of Deputies. Both occurred in the Social Security and Family Committee, which considers health-related proposals. Representatives of the Ministry of Health, civil society, academia and industry were invited. The first public hearing was held in October 2017 to debate a recommendation from the National Health Council proposing the adoption of fiscal policies to reduce the intake of sugar-added processed drinks and to increase the consumption of healthy foods. The second hearing was held in December 2018 to discuss PL 8541/2017 and its attached bills (PL 8675/2017 and PL 10075/2018), which aimed at increasing taxes on sugary drinks.

Two of the bills (PL 8541/2017 and PL 10075/2018) propose to increase two already existing taxes: a Value-Added Tax (*Imposto sobre Produtos Industrializados*) and a gross receipt tax (*Cofins*). The third (PL 8675/2017) proposes to create a new excise tax (*Cide-Refrigerantes*). The bills will be fully described elsewhere^(33)^. In general, there are slight differences among the bills with regard to the drink categories to be taxed and the thresholds on sugar content. It is important to point out that the legislative process is still ongoing in the first Committee of the first Legislative House, so the proposals may undergo significant changes.

Industry representatives were two prominent trade associations: Abir (Brazilian Association of Soft Drinks and Non-Alcoholic Beverages Industry) and Unica (Brazilian Sugarcane Industry Association). Abir represents Big Soda (among their associates are Ambev, Coca-Cola, Mondelez, Nestlé, Pepsi and Red Bull) and participated in both hearings; Unica represents the main sugar, ethanol and bioelectricity producers in the south-central region of the country and participated in the second public hearing only.

Video recordings and transcripts of the meetings are publicly available on the Chamber of Deputies website and were used for content analysis of the industry’s representatives’ arguments against the increase of sugary drinks taxation in Brazil. Coding and classification of arguments followed a framework designed to assess CPA strategies and practices of the food industry, which was developed by researchers who are part of INFORMAS, the International Network for Food and Obesity/Non-Communicable Diseases Research, Monitoring and Action Support^(14)^. The authors established that new clusters would be included whenever arguments not covered in the original framework emerged. The arguments are presented as examples, but they were not quantified, as they were collected on only two occasions (public hearings).

Data were collected and coded by ABM in August 2020 using NVivo 12^(34)^ and checked by APBM. Any disagreement on coding and classification of arguments was resolved by consensus. Extracts of representatives’ discourses were translated by a native speaker of English who is fluent in Portuguese and are presented in Supplementary File 1. The coding was conducted as follows: ‘A’ refers to extracts from the representative of Abir and ‘U’ to the representative of Unica; the numbers 1 and 2 next to ‘A’ codings refer to the occasion the argument was used (2017 and 2018, respectively). The last number refers to the sequence of the argument in their speeches.

## Results

We identified the use of ‘Information and messaging’ and ‘Policy substitution’ strategies to directly lobby legislators against sugary drinks taxation. The first strategy refers to the dissemination of information that favours the practices and activities of the industry and was used by both representatives. The second strategy refers to voluntary or self-regulation initiatives proposed by the industry when threatened by public regulation and was used only by the representative of the sugary drink industry. Table 1 shows the practices and mechanisms related to these strategies. Highlighted practices and mechanisms emerged in this analysis and were not covered in the original CPA framework.

**Table 1:** Lobbying strategies, practices and mechanisms used against sugary drinks taxation that emerged in public hearings in the Brazilian Legislature, October 2017 and December 2018

Strategies	Practices	Mechanisms	Abir	Unica
Information and messaging	Stress the economic importance of the industry	Stress the number of jobs supported and the money generated for the economy	Yes	Yes
	*Stress the environmental importance of the industry*	*Stress the reduction of CO*_*2*_ *emissions promoted by the industry*	No	Yes
	Promote deregulation	Highlight the potential burden associated with regulation	Yes	Yes
		Demonise the “nanny state”	Yes	Yes
		*Question the effectiveness of regulation*	Yes	Yes
		*Suggest public-private partnerships*	Yes	No
	Frame the debate on sugar- and health-related issues	Shift the blame away from the industry	Yes	Yes
		*Shift the blame away from the product*	Yes	Yes
		Promote the good intentions and stress the good traits of the industry	Yes	Yes
		Emphasise the food industry’s actions to address public health-related issues	Yes	No
		*Question sugary drink taxation as a public health recommendation*	Yes	No
	Shape the evidence base on sugar- and health-related issues	Cherry pick data that favour the industry	Yes	Yes
Policy substitution	Promote alternatives to policies	Promote voluntary codes	Yes	No
		Promote self-regulation initiatives	Yes	No

Regarding the ‘Information and messaging’ strategy, four practices described in the original framework were identified (‘Stress the economic importance of the industry’, ‘Promote deregulation’, ‘Frame the debate on sugar- and health-related issues’ and ‘Shape the evidence base on sugar- and health-related issues’). An additional practice emerged in this strategy: ‘Stress the environmental importance of the industry’, which was used by the representative of the sugar cane industryand had not been described in the original framework.

As for mechanisms not included in the original framework, we identified ‘Stress the reduction of CO_2_ emissions promoted by the industry’ under ‘Stress the environmental importance of the industry’ practice; ‘Question the effectiveness of regulation’ and ‘Suggest public-private partnerships’ under ‘Promote deregulation’ practice and ‘Shift the blame away from the product’ and ‘Question sugary drink taxation as a public health recommendation’ under ‘Frame the debate on sugar- and health-related issues’ practice.

Overall, arguments related to these practices and mechanisms refer to the number of jobs supported by the industry sector, and the revenues generated for the economy; a supposed role of sugar cane crops in reducing CO_2_ emissions; the burden associated with regulation, which would lead to (a) increased prices, causing negative impact on the whole industry chain and job losses and (b) increased tax burden, which is already elevated in Brazil; criticisms regarding the role of the state in regulating the private sector, which allegedly interferes in individual choices; questioning whether taxation is an effective policy for tackling obesity and diet-related NCDs; suggesting that the industry should work together with the government and civil society organisations; shifting the blame for obesity and diet-related NCDs away from both industry practices and the product (sugar and sugary drinks); promoting the good intentions of the industry and emphasising their actions to address the problem of obesity and diet-related NCDs; questioning whether international health organisations actually recommend increasing taxes on sugary drinks; and cherry picking data that favour the industry, casting doubt on the relationship between sugar and sugary drinks intake and the occurrence of obesity and diabetes, as well as on the actual share of sugar and sugary drinks in the Brazilian diet.

Concerning the ‘Policy substitution’ strategy, we identified under the practice ‘Promote alternatives to policies’ the mechanisms ‘Promote voluntary codes’ and ‘Promote self-regulation initiatives’, both reported in the original framework. Arguments related to these mechanisms refer to the adoption of voluntary codes to self-regulate marketing directed at children under the age of 12 years and food labels, as well as to the signature of a voluntary agreement between the industry, the Ministry of Health, and the Brazilian Health Regulatory Agency (Anvisa) to reduce the sugar content in processed foods.

Table 2 presents the arguments used under each of the mechanisms identified and the corresponding coded examples, which are shown in online Supplementary material, Supplementary File 1. Although the representative of the sugary drink industry uses a wider variety of arguments than the representative of the sugar cane industry does, both largely maintain a close alignment. The most pronounced differences were found in respect to the mechanism ‘Stress the reduction of CO_2_ emissions promoted by the industry’, which was only mentioned by the representative of the sugar cane industry, and the mechanisms and arguments related to the ‘Policy substitution’ strategy, only mentioned by the representative of the sugary drink industry.

**Table 2:** Mechanisms and related arguments used against sugary drinks taxation emerged in a public hearing in the Brazilian Legislature, October 2017 and December 2018

Mechanisms	Arguments	Examples		
		Abir		Unica
		2017	2018	
Information and messaging				
Stress the number of jobs supported and the money generated for the economy	The sector supports an elevated number of jobs	–	–	U.1
	The sector generates high revenues for the economy	A.1.2	–	U.1
*Stress the reduction of CO*_*2*_ *emissions promoted by the industry*	Sugar cane crops reduce CO_2_ emissions and help improve air quality in big cities	–	–	U.2
Highlight the potential burden associated with regulation	Taxation will increase prices of the product	A.1.18	A.2.21	–
	Taxation penalises society as a whole, especially the disadvantaged	A.1.16	–	U.16
	Sugary drinks taxation is discriminatory because it blames the product as the sole cause of obesity	–	A.2.2	–
	Regulation will have economic impacts on the whole industry chain	A.1.16; A.1.18	A.2.21	–
	Taxation will result in job losses	A.1.18	–	–
	Most Brazilians disapprove of the elevated amount of taxes already collected by the government	A.1.15	–	–
Demonise the “nanny state”	Taxing sugary drinks will cause impact on the free choice of individuals	A.1.8; A.1.20	A.2.10	U.16
	Most Brazilians believe the government should not interfere in individual choices	A.1.15	–	–
*Question the effectiveness of regulation*	Taxation has not been effective in other countries	A.1.17	A.2.18	–
	Other countries have rejected or withdrawn sugary drinks taxation	A.1.14	–	–
	Taxes on sugary drinks in Brazil are already high	A.1.13	A.2.18; A.2.20	–
	Taxing products does not promote health	–	A.2.3	–
	Taxation is not a proper solution	A.1.6; A.1.7; A.1.12; A.1.17	A.2.19; A.2.23	U.16
	Taxation does not address the problem of obesity and diet-related diseases	–	A.2.4	–
	Impact analysis of the policy must be carried out by the government before it is implemented	A.1.3	–	–
	Other types of government policies should be implemented	A.1.9; A.1.18; A.1.19	–	–
	It is unknown whether increased prices will reduce intake	A.1.20	–	–
*Suggest public-private partnerships*	Suggest partnerships between the industry, the government and civil society	A.1.19	–	–
Shift the blame away from the industry	Education and behaviour change are needed	–	A.2.23	U.15
	Unbalanced diet is a major problem	A.1.4	A.2.2	–
	Individuals have the right and responsibility to choose their own diet	–	–	U.3; U.4; U.8
	Nutrition education and counselling have not been properly carried out	–	A.2.6	–
	Obesity is a multifactorial disease	A.1.4; A.1.5; A.1.7	A.2.1; A.2.2; A.2.23	U.4; U.15
	Physical inactivity also plays a role in obesity	A.1.5	A.2.2	U.4; U.8; U.11; U.15
	Genetics and hormones also play a role in obesity	A.1.5	A.2.2	–
	Anxiety and depression also play a role in obesity	A.1.5	A.2.2	–
	It is the role of the government to inform the population and develop policies to address obesity	A.1.9	A.2.10; A.2.17	–
*Shift the blame away from the product*	Sugar is a source of energy	–	–	U.6; U.8
	Sugar is not the only sweetener high in calories	–	–	U.9
	The problem lies in excessive intake	A.1.4; A.1.28	A.2.6	U.4; U.6
	Any foods can be part of a balanced diet	–	–	U.4; U.8
	Sugar has cultural and historical aspects	–	–	U.3; U.4; U.5
	Sugar and sugary drinks are not the (only) cause of obesity	A.1.1; A.1.6; A.1.7; A.1.12	A.2.1; A.2.2; A.2.16	–
	Food processing is needed and not necessarily harmful	A.1.27	–	–
Promote the good intentions and stress the good traits of the industry	The industry is working to address obesity and diet-related non-communicable diseases	A.1.19; A.1.25; A.1.26	A.2.7; A.2.12	–
	The industry supports prohibiting the sales of sugary drinks to children under the age of 12 years in school environments	–	A.2.15	–
	The industry is working to spread messages on balanced diet and lifestyles	–	–	U.14
	The industry should be considered part of the solution	A.1.29	–	–
Emphasise the food industry’s actions to address public health-related issues	The industry has been working on product diversification	A.1.20; A.1.21	A.2.9; A.2.14	–
	The industry sponsors sports and physical activity programmes	A.1.22	A.2.13	–
	The industry is reducing the amount of sugar in its products	A.1.20; A.1.24	A.2.8	–
	The industry is reducing marketing targeted at children under the age of 12 years	A.1.22; A.1.26	A.2.11	–
	The industry self-regulates food labelling	A.1.23; A.1.26	A.2.17	–
	The industry has committed not to sell sodas to children under the age of 12 years in schools	A.1.23	–	–
*Question sugary drink taxation as a public health recommendation*	The World Health Organization and the United Nations have not recommended taxing sugary drinks	A.1.11	A.2.22	–
Cherry pick data that favour the industry	There is no clear relationship between sugar or sugary drinks intake and obesity or diabetes	A.1.12	A.2.5; A.2.6	U.10; U.11; U.12; U.13
	Sugary drink intake is low and has been decreasing in Brazil	A.1.7; A.1.8; A.1.10; A.1.13	A.2.4; A.2.6; A.2.18	–
	Sugar intake from processed foods is low in Brazil	–	–	U.7
Policy substitution				
Promote voluntary codes	The industry has signed a voluntary agreement to reduce the sugar content in processed foods	–	A.2.8	–
Promote self-regulation initiatives	The industry self-regulates its marketing directed at children under the age of 12 years on television	–	A.2.11	–
	The industry is working on the self-regulation of food labels	–	A.2.17	–

## Discussion

The results of this study represent an important initial step in understanding sugary drink and sugar industries strategies, practices and arguments to prevent, postpone or shape the adoption of public policies with the aim of reducing the impact of sugary drinks intake on the prevalence of obesity and other diet-related NCDs in the Brazilian population.

The framework by Mialon *et al*.^(14)^ describes a set of six strategies used by the food industry to influence public health policies. Although in this analysis we only found the use of two of them (‘Information and messaging’ and ‘Policy substitution’), these are likely to be the most easily applied in the context of a public hearing to directly lobby legislators. Different contexts might result in the identification of different strategies and practices. For instance, a study recently published by Serodio *et al*.^(23)^ points to the use of ‘Information and messaging’ and ‘Constituency building’ strategies by The Coca-Cola Company representatives when communicating with public health academics of Global Energy Balance Network. Ojeda *et al*.^(35)^ applied both the methodology to systematically identify CPA of the food industry and the framework to classify its strategies and practices to assess sugar-sweetened beverage industry attempts to influence public policy in Mexico. Based on publicly available information and interviews, the authors were able to identify the six CPA strategies.

In our study, the ‘Information and messaging’ strategy was used by the representatives of both associations. On the other hand, the ‘Policy substitution’ strategy seems to be more related to the sugary drink industry and thus has only been cited by the representative of Abir. The greater number of arguments used by the representative of the sugary drink industry possibly reflects a longer experience of the association in lobbying on health-related issues. There are no records of recent participation of the sugar cane association in other public hearings addressing health policies in either legislative House in Brazil (information requested by FOI legislation).

Mechanisms and practices that emerged in our analysis are quite consistent with those identified by Tselengidis and Östergren^(32)^ under the same strategies. This could be explained by the fact that commercial interests of transnational corporations are at stake. Research mapping the CPA of the ultra-processed food and drink industry has shown that they use similar strategies and practices to avoid regulation across different countries and settings^(36,37)^. In our analysis, we have also identified the mechanism ‘Question the effectiveness of the regulation’, which was used mainly by the representative of the sugary drink industry. We have also identified arguments promoting the good aspects of sugar and shifting the blame away from it and sugary drinks, which we classified under the argument ‘Shift the blame away from the product’. Therefore, in our study, arguments related to industry practices were regarded under the mechanism ‘Shift the blame away from the industry’ and the ones related to the product under ‘Shift the blame away from the product’. Arguments related to these mechanisms seem to be used in conjunction to cast doubt on the role of sugar and sugary drinks as well as that of industry practices on the development of obesity and diet-related NCDs. As a consequence, they also cast doubt on the proposed regulation.

One practice which had not been reported before was found in our analysis: ‘Stress the environmental importance of the industry’. The only mechanism used in it was ‘Stress the reduction of CO_2_ emissions promoted by the industry’, and it is likely to be found only in countries where sugar cane is the main source of sugars for the industry, such as Brazil, the largest producer of sugar cane worldwide. This argument is very much related to the sugar cane industry and not expected to be used by the sugary drink industry. In fact, replacing fossil fuels for sugar cane bioethanol might indeed reduce greenhouse gas emissions. However, increasing sugar cane crops raises environmental concerns related to its intensive use of water and deforestation^(38-40)^.

Although the use of arguments related to environment protection by the sugary drink industry was not identified in our analysis, this subject must also be brought to the discussion. Promoting actions such as sustainable packaging, bottle recycling campaigns and reduction on carbon footprints is just a smoke screen to divert attention from major environmental issues such as water pollution and scarcity^(41)^. Plastic bottles of both sugary drinks and bottled water are an important source of environmental pollution and are among the dominant type of oceanic, coastal and riverine debris. Plastic debris represents a global problem that not only impacts ecosystems and wildlife but also human health^(42-46)^. There is also concern about the sustainability of the intensive use of plastic, as its production requires large amounts of fossil fuels and water^(47,48)^. Therefore, the sugary drink industry is a significant part of the problem, not the solution for it.

We have also identified a mechanism not previously included in the original CPA framework under the ‘Promote deregulation’ practice, which we called ‘Suggest public-private partnerships’. The argument under it suggests that the industry, the government and civil society should work together to address the problem of obesity and other diet-related NCDs. It can be used not only as a way to promote deregulation by the government but also to push for the development of voluntary agreements such as the one signed by the most prominent ultra-processed food industry associations in Brazil, the Ministry of Health and the Brazilian Health Regulatory Agency (Anvisa) in November 2018^(49,50)^. This agreement aimed at reducing the sugar content in industrialised processed foods in Brazil, including some types of sugar-sweetened beverages (sodas, 50% fruit juices and powdered drink mixes). Notwithstanding, it was widely criticised by public health professionals and advocates. The targets established in the agreement were fairly modest, and the sugar content of most industrialised products was already within the limits set to be reached by the end of 2022^(51-53)^.

Most of the arguments opposing the regulation identified in our analysis resemble the ones reported by Hilton *et al*.^(54)^, who have assessed stakeholder discourse in The UK’s newspapers following the government announcement of a soft drink industry levy. Arguments identified by the authors state that: taxation would not result in behaviour change; a single nutrient should not be demonised; obesity is a complex problem, and a fiscal measure is not sufficient to resolve it; the public rejects additional taxes; obesity is rising despite the decline in sugary drinks intake; sugary drinks are not a significant source of dietary calories; a reduction in sugary drinks consumption would not have a significant impact on obesity and other diet-related NCDs; taxation would have negative impacts on the industry, on the economy and on consumers, especially those in lower-income groups; taxation is likely to cause job losses; the industry has a role in promoting public health; there is no need for taxation or other regulation because the industry is voluntarily reformulating its products. The arguments found in our study are also in line with several of the corporate claims identified by Fooks *et al*.^(55)^ when assessing industry submissions in response to the South African National Treasury’s policy paper on taxation of sugar-sweetened beverages. According to the authors, the collected evidence reveals the use of a ‘policy dystopia’ narrative, in which industry players declared that consequences of the tax would include, among others: an elevated number of lost jobs, business failures across the supply chain, disproportionate economic impacts on lower-income households and an insignificant impact on population health. Regarding population health outcomes, industry players argue that sugar-sweetened beverages represent only 3% of energy intake in that country; therefore, a decline in consumption of such products is unlikely to reduce obesity; as sugar consumption within the country was already declining, it could not be considered a driver of increasing obesity rates and sugar-sweetened beverages were likely to be replaced with other energy-dense products by consumers. Despite all these similarities, we draw attention to the use of arguments related to two mechanisms in particular which have not been reported in the previous studies we are aware of: the environmental claim that sugar cane crops reduce CO_2_ emissions and the questioning of sugary drinks taxation as a recommended public policy by international health organisations.

Our analysis of arguments against sugary drinks taxation in the context of a public hearing has made clear that strategies and practices are not carried out individually; rather they complement each other. First, participation in public hearings is in itself counted as CPA, under the ‘Information and messaging’ strategy and the ‘Lobby policy makers’ practice^(14)^. Second, participation of the industry in voluntary codes with the government and self-regulation initiatives, which are part of the ‘Policy substitution’ strategy, is used as tools to frame the debate, under the mechanisms ‘Promote the good intentions and stress the good traits of the food industry’ and ‘Emphasize the food industry’s actions to address public health-related issues’.

We should recall that assessing the validity of the arguments presented by the industry representatives was not under the scope of this analysis. However, there have been manifest contradictions between some of the arguments that need to be addressed. Despite casting doubt on the effectiveness of sugary drinks taxation to respond to the obesity problem, the representative of the sugary drink industry recognised in both hearings that implementing such policy would result in increased prices of the products – which is exactly the objective of the fiscal policy. Criticisms against state interventions on the economy and individual choice have been expressed, but the role of the government in promoting health has not been dismissed. On this basis, it must be highlighted that public health professionals and advocates do not endorse sugary drinks taxation as the one and only solution to obesity and diet-related health problems. On the contrary, sugary drinks taxation is advocated among a set of recommendations that should be implemented in combination. Besides that, we keep in mind that the role of the Brazilian government in developing and implementing public policies to protect, promote and improve the population’s health is well established in the Federal Constitution^(31)^.

Unfortunately, sugary drinks taxation has not been given priority in the health policy agenda 4 years after the commitment to the United Nations’ Decade of Action on Nutrition. So far, discussion of this public policy has been limited to the Social Security and Family Committee in the Chamber of Deputies, and no further actions such as public consultations, for instance, have been taken. Because lobbying is not regulated in Brazil, the only publicly available information on arguments undertaken by the sugar cane and sugary drink industries to directly influence lawmakers is limited to participation in public hearings. Although the strategies and arguments put forward behind the scenes are not known, there is evidence that the sector is highly politically active. Representatives of trade associations frequently have a seat in public hearings in both legislative Houses to discuss health and nutrition-related issues, and the ultra-processed food industry has a relevant history of electoral campaign contributions^(56)^.

As a matter of fact, a brief analysis of the chain of events that lead to the second public hearing suggests a strategy to postpone deliberation and possibly result in non-decision, as the 55th Congress would end in February 2019 and bills on which no decision had been made would be archived. On 6 November 2018, the rapporteur of the PL 8541/2017 and its attached bills (PL 8675/2017 and PL 10,075/2018) presented a favourable report, recommending their approval in the Committee. That meant deliberation could occur any time soon. However, on 27 November 2018, a Federal Deputy member of the Committee requested a public hearing and on 28 November 2018, two Federal Deputies (one member and one non-member of the Committee) requested the bills be removed from the deliberation calendar. The three Federal Deputies involved in the latter events had a history of campaign contributions from Big Soda. According to publicly available data from the Superior Electoral Court, all of them had received contributions from companies related to Coca-Cola and one of them also from Ambev. Therefore, we cannot rule out the possibility that the lobbying activities of this highly organised and economically powerful special interest group might be hindering the efforts in the Brazilian Legislature to implement public policies aimed at tackling obesity and other diet-related NCDs^(30,57)^. We must highlight that by March 2021, no decision has been made. The bills are still awaiting deliberation in the first Committee of the first legislative House. As a result, an enactment should not be expected any time soon.

Limitations of this study should not be overlooked. The arguments presented herein are likely to be underestimated, as the analysis is limited to two public hearings and a public policy to tax sugary drinks has not been widely debated by the Brazilian government so far. In addition, strategies, practices and arguments that are possibly being adopted outside the Brazilian Legislature are not taken into account. Finally, lobbying arguments shown in this study are limited to those put forward by trade associations, as no individual industry actively takes part in public hearings in the Brazilian Legislature. As there is evidence that the ultra-processed food and drink industry acts politically mainly through trade associations^(57-59)^, arguments reported in this analysis are very likely to represent the whole sector.

In conclusion, the arguments against sugary drinks taxation identified in this study are related to the ‘Information and messaging’ and ‘Policy substitution’ strategies. Overall, representatives of the industry sector claim such type of regulation would result in economic losses, question the role of the government with regard to the development of public policies to protect citizens’ health and cast doubt on scientific evidence on the role of sugary drinks in disease and the effectiveness of sugary drinks taxation as a public health policy. The much questioned ‘role’ of the industry in addressing public health-related issues is reinforced, and the sugar cane industry brings in allegations related to environmental protection. Although arguments used by the sugary drink industry appear to be more refined (and possibly also their lobbying strategies with regard to sugary drinks taxation), we cannot dismiss the economic relevance and the political power of the sugar cane industry in Brazil.

At last, it should be borne in mind that the CPA of sugary drink and sugar cane industries in Brazil is not limited to what has been shown in this research. Our results represent only the tip of the iceberg: the strategies, practices and arguments used by two of the strongest opposers to the regulation in the country during public hearings. Additional research should be carried out to address whether and under what conditions lobbying from this industry sector is effective in the Brazilian Legislature.

## Supplementary material

For supplementary material accompanying this paper visit https://doi.org/10.1017/S136898002100149X. click here to view supplementary material
