# Supplementary material for: Sugary drinks taxation: industry’s lobbying strategies, practices and arguments in the Brazilian Legislature
Source: Public Health Nutr. 2021 Apr 6;25(1):170–9. doi: 10.1017/S136898002100149X (PMC8825954; doi:10.1017/S136898002100149X)
Supplement: Supplementary file 1 [file S136898002100149Xsup001.docx]

**Supplementary File** Extracts from the discourses of representatives of sugary drinks and sugar industry associations in public hearings in the Brazilian Legislature.

| **ABIR, October, 2017** |
| --- |
| **A.1.1** ‘Firstly, I would like to make it clear that we are not here to discuss sugar, in its over-consumption, if it is good or bad, or if it makes people put on weight or not. The public hearing is to discuss a taxation policy and I shall adhere to this. However, it is important to clarify at the outset that it is not the consumption of soft drinks which is the cause of obesity, especially in Brazil. This is the tone that I would like to demonstrate and ensure that you gentlemen understand.’ |
| **A.1.2** ‘Our sector comprises manufacturers of all ranges and sizes, whose numbers are worth mentioning. We have a large number of companies, factories, market share, volume and investments. In 2015, for example, the amount of 7 billion Reais, and we collected 10 billion Reais [*Brazilian currency*] in taxes, Mr. Mombelli, 10 billion Reais [*Brazilian currency*] in annual taxes. I shall demonstrate herein that taxation is not minimal, contrary to what has been put forth.’ |
| **A.1.3** ‘(…) Yes, it is also necessary to assess the impact of a public policy’. This economic impact is very important. It is not possible to attack or attempt to create fiscal policy or developmental policy without analyzing the whole picture.’ |
| **A.1.4** ‘Everyone has said here (…) that obesity is multifactorial. It is said here that the main cause of obesity is an unbalanced diet, or rather, excess in food and sugar intake.’ |
| **A.1.5** ‘Besides this, there is the issue of sedentarism. A map has been brought here showing overweight in all the states, from north to south, showing exactly sedentary lifestyles versus unbalanced diets, which counts largely towards the issue of obesity itself. Beyond mentioning genetic and hormonal factors, which count, there are the issues related to the present day, the busyness of modern life, globalized lives, jobs, work, problems with mobility in traffic, and there is the issue of anxiety and depression as well.’ |
| **A.1.6** ‘It is not the soft drink, Ladies and Gentlemen, Deputies, which is the main cause of obesity. There is the intention here to create an unpopular discussion, perhaps populistic, easily attacked, that attacking and overtaxing soft drinks will resolve the problem of obesity. Soft drinks are not the villains, and we shall demonstrate this with numbers.’ |
| **A.1.7** ‘Furthermore, I shall say this in advance, that the intention here is to create discrimination, Deputies. They want to use the soft drink, whose numbers show that its consumption is low, without analyzing all multifactorial issues [*related to obesity*], and tax it, and in so doing resolve the obesity problem. Moreover, what about the rest, such as our coffee, in which we put sugar, our unbalanced diet, our sedentary lifestyle? Are we also going to tax those who do not do exercise? Will this Congress overtax those who consume more than two thousand calories per day? Will we overtax those who suffer from stress at work and become obese? Will we label someone who is born with different genetic factors and with a tendency towards obesity as ‘ultra-processed’? This is an important point for you Deputies to analyze. In fact, it is not the sugary drinks that are the main problem, and they should not be the only ones being attacked.’ |
| **A.1.8** ‘By calculating the per capita consumption of soft drinks and industrialized juices in Brazil, and the energy content of such drinks, according to data collated by Nielsen, we can determine the caloric intake corresponding to the daily consumption of sugary drinks by Brazilians. Obviously, there are Brazilians who consume far more, and it is possible that there are those who consume less, but this is the average of the numbers. This is mathematics and statistics. It is important to consider the following: four percent of calories that we ingest come from soft drinks in Brazil. Are we going to overtax the soft drinks in Brazil? Are we going to take away the freedom of choice from our citizens? It is a constitutional principle of each citizen, of each human being. The power of choice, as long as they are informed.’ |
| **A.1.9** ‘The government must act so that there are public policies to this end, and not simply make the issues prohibitive. It doesn’t make sense.’ |
| **A.1.10** ‘According to data that the Government itself publishes, from VIGITEL [*a national survey*], obesity has increased over the last 10 years. It has gone from 11.8% to 18.9%, while there is another curve showing a 20% decrease in soft drink consumption.’ |
| **A.1.11** ‘There is a recommendation by WHO, which is not really a recommendation, as there is no scientific basis whatsoever. I state this, and bring forward to the current scenario 25 or 30 possible issues with which the Government should be concerned. And the Brazilian Government concerns itself firstly with taxing soft drinks. It does not wish to discuss the multifactorial issues; it does not wish to discuss physical exercise policy; it does not wish to discuss, in Congress, the adoption of an effective public policy against obesity. No, it wishes to tax soft drinks.’ |
| **A.1.12** ‘We have here a graph that we drafted based on the two graphs from VIGITEL [*a national survey*]. If obesity is increasing and soft drink consumption is decreasing dramatically, are we going to tax soft drinks? And what of other products, other foods, the multifactorial issues? It doesn’t resolve things, Ladies and Gentlemen.’ |
| **A.1.13** ‘This is the tax burden on soft drinks in Brazil: 40% of which is consumed in the can is taxed. And here we are discussing tax increase? We have many examples. Moreover, I shall start by citing, Ladies and Gentlemen, examples of what has been attempted worldwide. The emblematic example is Mexico. We are Latin; we are similar to the Mexicans. We are so similar to the Mexicans, with all due respect, that they consume six times more soft drinks than Brazilians do. The Mexican per capita consumption, Mr. President, is six times higher than that of the Brazilian. And there, the tax burden increased and reached, with the sugar tax, 28%, while ours is 40%. In other words, they consume six times more and the tax burden is 28%, and we consume six times less and our tax burden is 40% - and there is still the intention of a surcharge.’ |
| **A.1.14** ‘In Germany, this initiative was rejected for exactly this reason; in Australia, its own Health Ministry is against it; in Colombia it was rejected; in Denmark it was implemented in 2011 and withdrawn in 2012 – it cost five thousand jobs and did not reduce obesity; Great Britain will implement a policy and test it out.’ |
| **A.1.15** ‘Data from IBOPE [*a Brazilian polling organization*], relating to 2017, show that 90% of the population disapprove of Government taxation – it applies taxes where it should not; and on the tax payment scale, unfortunately the underprivileged are those who pay more; 94% believe they pay too much tax or more than they should. Data from DataPoder360 [*another Brazilian polling organization*], relating to October, 2017, show that 67% believe that the Government interferes a lot in people´s day-to-day; 78% believe that the Government should provide information, but people should be responsible for their choices. There is an aspect here, the work the industry has been doing regarding nutrition labelling.’ |
| **A.1.16** ‘There is a brutal impact on the production chain. Taxation already in place, of 40%, and still wanting to increase it, as discrimination against soft drinks and sugary drinks, is completely ineffective. Besides the impact on the chain – farmers, industry, distributors, sellers and consumers – taxation in Brazil, we know, is regressive and not progressive. Those most penalized will be, or would be, those with low income; they are the ones who will suffer the most with this. And many times they may be consuming more out of necessity.’ |
| **A.1.17** ‘The international experience, some examples I have already cited, shows that taxes do not contribute to absolutely anything in relation to this. In other words, such a measure was not effective, is not effective. A study by the McKinsey Global Institute, for example, concluded (…) that no isolated action has a significant impact. Taxing sugary products is one of the least effective initiatives in the fight against obesity.’ |
| **A.1.18** ‘The WHO says we must consume healthy foods; do physical exercise; integrate and reinforce pre-natal care in order to avoid the genetic issue; monitor diets during childhood; recommend diets in schools where there are overweight families; many other measures; and perhaps tax sugary drinks. Then, in the first discussion that we have, we will try to resolve the problem the easy way: increase the burden on the production chain, increase the burden on the consumer, generate unemployment and create impact.’ |
| **A.1.19** ‘I have already said, many times, that the industry wishes to join in the fight against obesity. It is already doing this, but unfortunately, it does not appear so. (…). The Government, together with this Congress, have an important role, from investing in nutrition education, to encouraging physical exercise, and developing actions in favor of a healthy lifestyle. We have the opportunity to create a partnership between the industry, the public sector and civil society. |
| **A.1.20** ‘What has the industry been doing? In recent years, there has been a reduction in the quantity of sugar, and there have been many, many actions in order to reach the desired results. Such as portfolio expansion, with new options and with different caloric contents in teas and soft drinks. The industry, thanks to technology, has the capacity to provide consumers with options. It invests and provides the option of products with and without sugar, and with an intermediate amount of sugar, etc. Will the freedom of choice be taken away by increasing the price? The fiscal policy that is proposed means increasing the price, taxing soft drinks. The Government will collect more - and I have my doubts whether this revenue will be allocated to some type of fund against obesity, by today’s existing policies – and if it will discourage consumption, it will increase the price of the product, by means of taxation, taking away freedom of choice, since consumers will have the option of a sugar-free drink.’ |
| **A.1.21** ‘We also have a variety of sizes. That is to say that in the packaging list, the size of the packaging has a strong influence, for instead of buying four liters or two liters, the consumer can buy 200 milliliters, 300 milliliters, 350 milliliters, or 400 milliliters.’ |
| **A.1.22** ‘There is investment in sport and the discontinuation of marketing targeted at children. This is not discussed. ABIR has made a commitment with all the companies to prohibit advertising aimed at children.’ |
| **A.1.23** ‘And we also have a policy of not selling soft drinks at schools to under 12s, and we are working on labels to inform if there is, in fact, excess of sugar.’ |
| **A.1.24** ‘The issue of sugar reduction is extremely important. The sector has already reduced, in recent years, 11% of sugar in its products – I am not referring to creating new options; the sector has already reduced the use of sugar – and presented recently to the Ministry of Health a proposal for further reduction.’ |
| **A.1.25** ‘Our association is building a general commitment, not only in relation to soft drinks, but also in relation to sugary drinks in general. The main companies have already taken this initiative, and the small companies are still without a portfolio to substitute this offer, without leaving this important market. Therefore, we still do not have a full commitment from the association and the whole industry, but we are on this track. However, it is not only about soft drinks.’ |
| **A.1.26** ‘We represent the beverage industry. We understand that there is a non-social responsibility to tackle obesity, as we have done so with the unanimous commitment related to marketing targeted at children. There is no more advertising targeting the children’s market. With regard to the issue of drinks in schools, we are working on a labelling system to improve information provided and we have already made reductions in sugar content, as I have shown here.’ |
| **A.1.27** ‘What must not happen is to avoid technology. Globalized life and the consumption have required practical alternatives. This cannot be condemned if there is safety, or in other words, processed does not mean bad; in fact, it may be safe. The issue relates to eating habits and everything that has been put forth.’ |
| **A.1.28** ‘The excess of soft drinks, the excess of sugar, the excess of salt, the excess of juice, the excess of meat, all excess is what unbalances this.’ |
| **A.1.29** ‘The industry is part of this process. We are doing everything possible to implement this. However, the industry must not be trivialized and criminalized.’ |
| **ABIR, December, 2018** |
| **A.2.1** ‘(…) an issue brought forward by all the participants who preceded me, Deputies, refers to the multifactorial issues of obesity. We are in agreement. Do not come here and present any argument that it is not an endemic problem in Brazil and worldwide, Deputies. Obesity exists, has to be treated, and has to be tackled. The question is: are soft drinks the only cause of obesity in Brazil?’ |
| **A.2.2** ‘If new taxation were created, we would be talking about, in first place, of a discriminatory taxation. I believe this should be mentioned. Regardless of fiscal issues that we shall get into, it is important to point out this issue: are soft drinks the cause of obesity in Brazil? We have an unbalanced diet, sedentarism, genetic factors, anxiety and depression. As a whole, an unbalanced diet, cited here by the Ministry of Health, is in fact one of the biggest problems.’ |
| **A.2.3** ‘I agree entirely with everything that has been said by the Ministry of Health, as I agree entirely with what the ACT [*ACT Promoção da Saúde, an NGO which advocates for health-related issues, including the right to healthier diets*] has said about the need to tackle obesity together. However, I disagree with the taxation, because I believe taxes do not produce health.’ |
| **A.2.4** ‘If we considered the volume of liters per capita consumed in Brazil, according to the number of inhabitants we would see that not even four percent of the number of calories are consumed in sugary drinks by Brazilians, which we are discussing in this public hearing. This is the result of a simple comparison of data. Therefore, this issue is not that relevant. However, I would like to emphasize that it is not relevant as a whole, but it is clear that excess can contribute. Will we overtax the excess discriminatorily, penalizing the price, or shall we take care of the problem?’ |
| **A.2.5** ‘There is a very interesting aspect here, mentioned by the Ministry of Health: with data from the VIGITEL system, already brought forward by UNICA in other examples, such as the Australian paradox, the issue of sugar intake has been decreasing in the United States. However, obesity continues on the rise. It has also been presented here the example of Canada, who is reducing the intake of sugar, but obesity and diabetes continue to rise. Therefore, it is not about an issue of contemporaneity, which only now has begun to decrease, but the effects that will come later.’ |
| **A.2.6** ‘In Brazil’s case, the consumption of soft drinks and sugary drinks has decreased in the last 10 years, from 30.9% to 14.6%. However, obesity has increased from 11.8% to 18.9%. Are soft drinks the cause of obesity? Although the Ministry of Health has put in place – I understood the purpose – I believe it is correct that counseling and education may be lower, but perhaps this has happened because counseling and education are not being carried out well. We cannot say that counseling, education and the cultural issue in the consumer’s mind are not relevant factors.’ |
| **A.2.7** ‘What must the industry do? The industry is not static; it is giving its best to reach this point.’ |
| **A.2.8** ‘A few days ago, we made a worldwide unprecedented agreement with the food industry. The food and beverage industry, with the backing of the Federal Government – in other words the Ministry of Health, with the participation of ANVISA – celebrated an agreement to reduce, under monitoring, the amount of sugar in sugary drinks and in other foods in the next four years. It is a voluntary agreement, guided by the same issues, same concepts, same principles and same goals carried out four years ago in the case of sodium. This shows that the industry does not want to be regulated or trampled upon. It is unfair, therefore, to say that the industry is static, and that it is doing all this to grow and is ignoring the reality. This is a recent example of what the agreement intends to reduce, more than what has been reduced in recent years; more than 144 thousand tons of sugar in the next four years, besides everything else the industry has done. It has expanded its portfolio.’ |
| **A.2.9** ‘It is up to the industry to provide options to the consumer. There is the sugary drink, the sugar-free drink, non-sugary drink, and water. The industry is expanding its portfolio and providing further options. We have never had such a large number of options on the supermarket shelves for Brazilians to choose from.’ |
| **A.2.10** ‘We cannot think that we are going to have a ‘Nanny’ State. The state must provide resources for information and acculturation, regulated by public policies so that the citizen has the right to choose.’ |
| **A.2.11** ‘I want to make it clear that I am fully in agreement with the issue of tackling childhood obesity. This is what we, at ABIR, did two years ago, a self-regulation of marketing aimed at children. We do not carry out any type of marketing, such as on open and cable TV, for those under 12 on our association products. On the basis of what? On the basis of international practices that I have been repeating. This initiative has been effective. Eighty-two percent of companies have complied with the first monitoring done by KPMG [*a private audit firm*] in 2017.’ |
| **A.2.12** ‘The industry is reducing sugar, and is reducing marketing aimed at children, just as it is investing in sports.’ |
| **A.2.13 ‘**Some time ago, we participated in a public hearing in this House. An interesting bill aimed to prohibit all types of publicity promotion or sponsorships on the part of the beverage industry in favor of Brazilian sports. The beverage industry is who most sponsors and promotes physical activity! It is obvious that the companies can exploit a bit of marketing, but this has already been much regulated, including by the National Council of Publicity Self-regulation, etc., and it has worked.’ |
| **A.2.14** ‘(...) there is the issue of various sizes. You may remember that we had options. In the past we had a one-liter bottle and a 250 milliliter or 350 milliliter can. Today, there are all sorts of sizes, for all preferences and occasions. They go from a 200 milliliter can to two-liter PET bottles. In the past, in my childhood, the size considered ‘family-sized’ was one liter, but increased to two liters, etc. Either families are increasing, or actually, we are drinking more soft drinks. The fact is that this must be discussed.’ |
| **A.2.15** ‘Furthermore, there are other movements that have been carried out regarding soft drinks in schools. There is an argument – there is also a bill that we support here, with the proviso of an age cut-off – regulating the sales of sugary drinks to under 12s. There is a bill, which intends to prohibit sugary drinks to basic education students as a whole. However, basic education goes up to 18 years old.’ |
| **A.2.16** ‘Yet how is this going to be controlled? How are the brands going to control the popcorn vendors or the cafeteria vendors who sell them? With all due respect – and I do not wish to stoke the fire here – it is worthless to discuss all these issues of marketing aimed at children, foods at schools, the issue of reducing sugar, shall we say, if there is no parallel control on the part of the State on education. Is it worth not selling soft drinks at schools, yet sell snacks made without knowing with which oil they were made? This a multifactorial issue. Soft drinks are not the grand cause.’ |
| A.2.17 ‘Another point is the issue of labelling. Labelling is not slow and is in motion. The industry itself is already ahead on this issue, carrying out self-regulation. We support a system that is not a system that, shall we say, villainizes the product. It warns. We support the issue of colors. This shall be discussed at the Brazilian Health Regulatory Agency (ANVISA). I believe that next year a public consultation will be carried out. We are in a hurry here; we wish to define this. We want so that the State provides information, regulations. Also, in this Congress, there are various bills following the same suit, independent of this concurrent, legislating, regulatory purview, between Congress and the regulatory body. Things are in motion, the industry is effectively doing its role to mitigate, and is working against obesity for sure. It is not static. |
| **A.2.18** ‘When the issue of taxation comes up, there is always a comparison with other countries. It is worth mentioning a practical example. It is always said that in Latin America, we have Mexico, which has also created the sugar tax. Look at the difference. Brazil today has the highest tax burden on soft drinks in Latin America, it is one of the highest in the world. We have today 36.9%, and this is without the sugar tax. Mexico, with the sugar tax, put into place two years ago, increased to 27%. I just want to make the comparison. Yet there is a difference worth highlighting: I do not know if you Gentlemen know, that in Mexico soft drinks are four or five times more consumed than in Brazil. It is a completely different situation. They have few water sources as well. There’s a lack of water, including for hydration. However, for the issue at hand it hardly matters. The fact is the following: in Mexico it didn’t cause effect.’ |
| **A.2.19** ‘I believe, and I have no doubt – and I would like to make it very clear, Deputy Paulo Teixeira, the bill’s author, and its rapporteur, Deputy Dr. Jorge Silva – that your intentions are commendable and meritorious. The intention is not simply to collect taxes. There is the intention to restrict consumption, and this is legitimate to Congress. We are here with the aim to debate, and not to make empty criticisms. That is why we are here. I admire the initiatives and attempts. I want to show you Gentlemen that taxation, in our understanding, is not the issue at hand.’ |
| **A.2.20** ‘In opening up the issue of taxes, it is worth putting forth that, for example, making the same comparison with Mexico, as we did, with 36.6% of sugar tax and 27.9%, the first tax imposed is the IPI [*Tax on Industrialized Products*]. With reference to the tax burden in the states, for example, we raise the issue of indirect taxes, which in Brazil reach 23%. This is the state tax, and recently the Brazilian states have gone further, with authorization from the Federal Constitution (…) and have increased two percentage points to complement the National Fund to Combat Poverty. It is worth saying that soft drinks are already being overtaxed to compensate certain things.’ |
| **A.2.21** ‘As we have also already mentioned, any and all taxation will affect a production chain. I am not preaching or giving a single perspective in the sense that there will be high unemployment, etc., but it does affect, it affects the industry entirely. The production chain will be affected, prices will rise. This was felt in all the countries where the sugar tax was imposed, effectively raising the prices. There is a study by Mackenzie [*a private University*] that says that this is not an isolated action.’ |
| **A.2.22** ‘I would like to call attention to, as the last relevant point, to the issue of the World Health Organization - WHO, of which we have much discussed here. I am showing a trajectory – I am not sure if you Ladies and Gentlemen can see – from 2017 to 2018. With regard to this text by the WHO, they are saying that it is a recommendation. With all due respect, this is not true. The WHO wanted to approve and implement in the United Nations a text of recommendation. The Minister of Education of Brazil was present at the United Nations in New York in September, 2017, and there was no consensus about placing soft drinks as best buys, and much less in overtaxing. There is a document that has been brought here and has been placed, shall we say, as a new document, from 23 November, which has not been approved. It was presented and is being discussed in Geneva, in an attempt to reopen the subject, with all the necessary diplomacy, in order to decide if it should be recommended or not. However, there is no document of approval by the United Nations regarding this. The WHO, in June, whose document I am placing on the slide, recognized that sugary drinks taxation is not a measure with approved effectiveness. The WHO said this in its document of June, 2018, or rather, withdrew their plea of recommendation because this had not been verified.’ |
| **A.2.23** ‘In conclusion, I state that we need to bring forth the issue of education. We need to treat this with its multifactorial issues. We believe, us in the industry, that the issue of taxation would not be the appropriate method. Why? Because taxes do not produce health.’ |
| **UNICA, December, 2018** |
| **U.1** ‘We are talking about a market that today has 370 productive units in around a thousand Brazilian cities. That is to say that we are present in more than 20% of Brazilian cities, with 70 thousand rural producers, generating almost one million direct jobs. The gross value of the chain is in the region of 100 billion Dollars, the GDP is 40 billion Dollars and the revenue in foreign exchange is in the region of 12 billion Dollars per year.’ |
| **U.2** ‘From the environmental point of view, the sector is today responsible for almost 16% of the Brazilian energy matrix. It is the second source [of energy] following hydroelectrics. We were responsible for the reduction of more than 600 million tons of CO_2_, a greenhouse effect gas, since the beginning of PROÁLCOOL [*a Brazilian Program aiming to reduce the use of fossil fuels*], in the mid-1970s. Moreover, the sector contributes significantly towards the air quality in the large cities, with the use of ethanol, which, according to the University of São Paulo, is responsible for avoiding around 1,500 deaths in the city of São Paulo per year, and 10 thousand hospitalizations caused by cardiovascular and respiratory diseases.’ |
| **U.3** ‘Quero iniciar a minha apresentação propriamente dita trazendo uma frase que consta do relatório da Organização das Nações Unidas para Alimentação e Agricultura — FAO a respeito de alimentação adequada: ‘Direito ao acesso regular, permanente e irrestrito à comida que corresponda às tradições culturais daquela pessoa e que assegure seu bem-estar físico e mental, respeitando a sua dignidade’. A nossa avaliação é a de que esse conceito traz a importância do acesso à alimentação, mas também ao direito do indivíduo a uma alimentação que corresponda às suas tradições culturais e o direito à livre escolha.’  ‘I would like to begin my presentation by stating a sentence from the report by the Food and Agriculture Organization for the United Nations – FAO - with regard to adequate diets: “The right to regular, permanent and unrestrictive access to food which corresponds to traditional cultures of that person and assures their physical and mental wellbeing, respecting their dignity.” Our evaluation is that this concept brings the importance of access to food, but also to the right of the individual to food, which corresponds, to their cultural traditions and the right to free choice.’ |
| **U.4** ‘This is the concept brought forth by Dr. Daniel Magnoni, from the Dante Pazzanese Cardiology Institute: that the universe of eating is a combination that involves the history and culture of the individual and their country; a balanced diet, with various types of food, including industrialized foods, with conscientious intake; and finally, the aspect of physical exercise versus the issue of sedentarism. We are talking about a multifactorial concept, which is related to obesity and chronic non-communicable diseases. Obesity involves excessive food intake, which combined with sedentarism generates imbalance between ‘calories ingested and burned’. However, obesity is multifactorial and can be influenced by genetics, endocrinal conditions, stress, sleeping problems, medication, among other aspects. The multifactorial aspect involves all these issues.’ |
| **U.5** ‘(…) sugar is part of Brazilian cultural tradition. The product has been present in the country for at least 486 years. It was brought here by Martim Afonso de Souza in 1532, and since then it has been part of our tradition throughout almost five centuries.’ |
| **U.6** ‘We understand that sugar is a source of energy essential to the human metabolism. It is accessible, from the economic point of view, and the main source of energy for the brain. The problem is not the intake, but excessive intake.’ |
| **U.7** ‘There is also an important piece of information: according to the Brazilian Household Budget Survey by IBGE [*Brazilian Institute of Geography and Statistics*], the industry would be responsible for less than 20% of sugar consumed by Brazilians annually. The other 80% are distributed among the sugars present naturally in foods, and especially among sugars added in the home.’ |
| **U.8** ‘The ideal is to opt for a balanced intake of sugar, as a source of energy, giving the consumer the right to choose, maintaining a balanced diet and doing physical exercise regularly.’ |
| **U.9** ‘We see the difficulty in measuring the intake of sugar. For example, when we compare one tablespoon of sugar to a tablespoon of honey. The tablespoon of sugar contains 15 calories and the tablespoon of honey 21 calories. It is difficult to take this type of concept to the consumer’s day-to-day.’ |
| **U.10** ‘I would also like to bring forth a very interesting aspect, called the Australian Paradox, which is nothing but the search for a correlation between sugar intake and the prevalence of obesity. I call attention to this graph. The red line corresponds to obesity and the blue line corresponds to sugar intake. We see a clear decrease in sugar intake in this country, and at the same time you can see an important increase in obesity, which takes us to reflect on the effective role sugar plays on obesity and other non-communicable diseases. Also, to really think about the importance of a multifactorial assessment when discussing the issue.’ |
| **U.11** ‘Here is the data relating to the United States. We can see, in blue, the intake of sugar decreasing 15% since 1999, and at the same time, we see an important increase in obesity in the American population. In this other graph, also related to the United Sates, we can see a phenomenon from 1980 onwards: the sugar intake increases until the mid-90s, then falls sharply, but obesity continues rising. In other words, once again we are talking about a process, which involves dietary habits and physical exercise. In short, it is about how the individual deals with food intake and exercise on a daily basis.’ |
| **U.12** ‘Here we see the same process in Canada. This thick line represents the rising cases of diabetes in Canada; the dotted line represents obesity; the green line represents sugar intake.’ |
| **U.13** ‘The data from these various countries show how important it is to have a multifactorial assessment. While obesity increased more than 300% in these countries in two decades, the per capita refined sugar intake fell or remained stable. In the cases that we have seen, it sharply fell.’ |
| **U.14** ‘(…) UNICA has also worked on a line of communication and promotion on a message of balanced intake. We have created a campaign called Sweet Balance [*Doce Equilíbrio*]. It is an initiative that intends to balance the public debate about the subject of sugar intake in Brazil. That is to say, to avoid vilification which we have seen and try to bring a balance to this debate, defending sugar intake as a source of energy in addition to wellbeing within a healthy lifestyle in physical and emotional aspects, where food is the fruit of personal choices and also a pleasure. We have created work fronts in this campaign. After a diagnosis, we will map and generate techno-scientific content and influencer engagement. It is a campaign that helps clarify to the population the importance of balance, which we call Sweet Balance [*Doce Equilíbrio*].’ |
| **U.15** ‘We understand that lifestyles depend on cultural habits, making it necessary to raise awareness and to provide strong, positive stimuli for effective changes. In other words, an entire behavioral change. Obesity is a grave problem that needs to be faced by society, but as a multifactorial problem. There is no way to close your eyes to a reality; almost two-thirds of Brazilians simply do not do physical exercise.’ |
| **U.16** ‘Taxing sugary drinks economically penalizes society for the creation of one more tax that society needs to pay for, especially for those less fortunate, who are most impacted in their right to choose. In addition, it is not an adequate solution to tackle obesity and other non-communicable diseases.’ |
